# Supplementary material for: DMRforPairs: identifying Differentially Methylated Regions between unique samples using array based methylation profiles
Source: BMC Bioinformatics. 2014 May 15;15:141. doi: 10.1186/1471-2105-15-141 (PMC4046028; doi:10.1186/1471-2105-15-141)
Supplement: Additional file 2 — DMRforPairs output for the comparison of A431-MCF7 and NA17018-NA17105. Please start from the HTML files in each folder. Available via the BMC Bioinformatics website. [file 1471-2105-15-141-S2.zip › 1394847754114233_MOESM2_ESM/A431_MCF7/figures/7505.pdf]

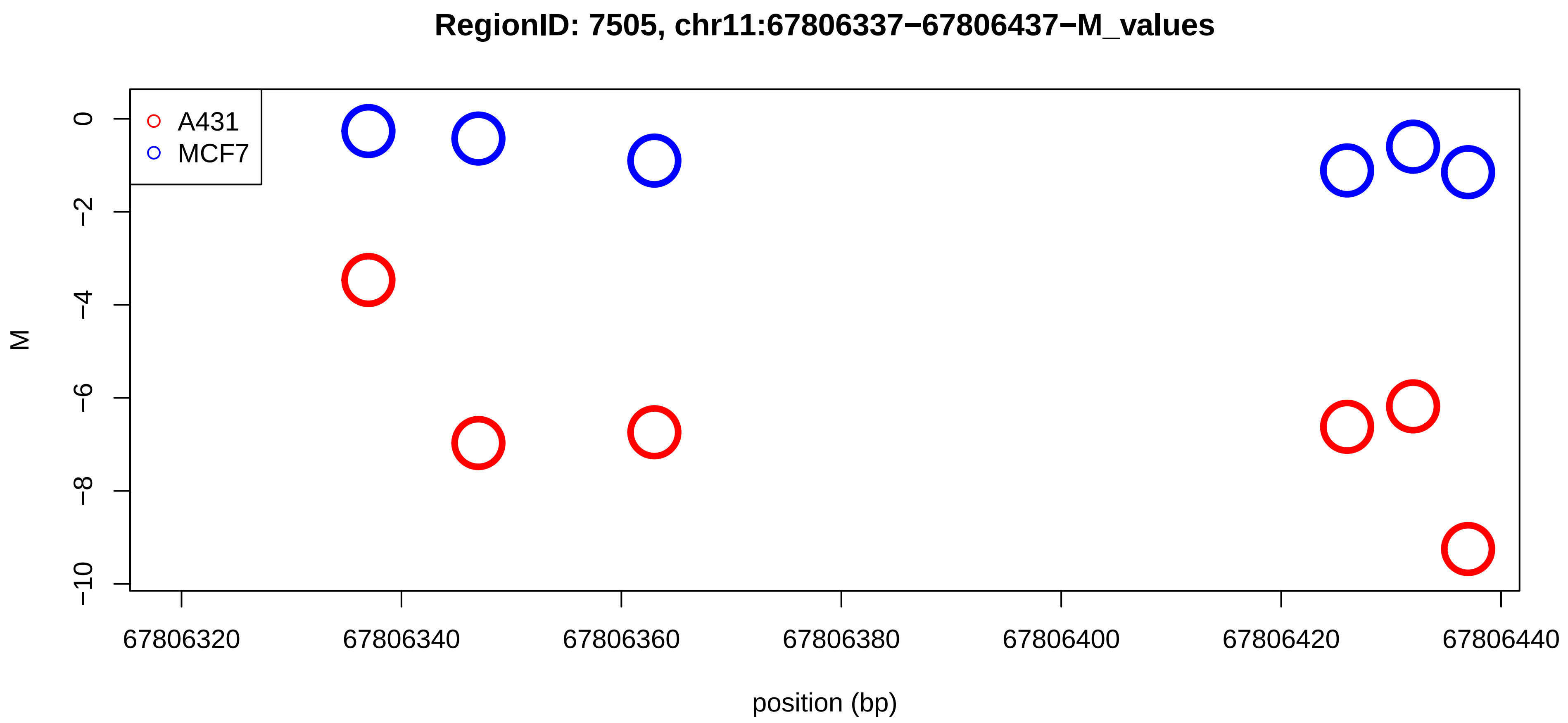
RegionID: 7505, chr11:67806337−67806437−M_values
A431
MCF7
M
0
−2
−4
−6
−8
−10
67806320
67806340
67806360
67806380
67806400
67806420
67806440
position (bp)

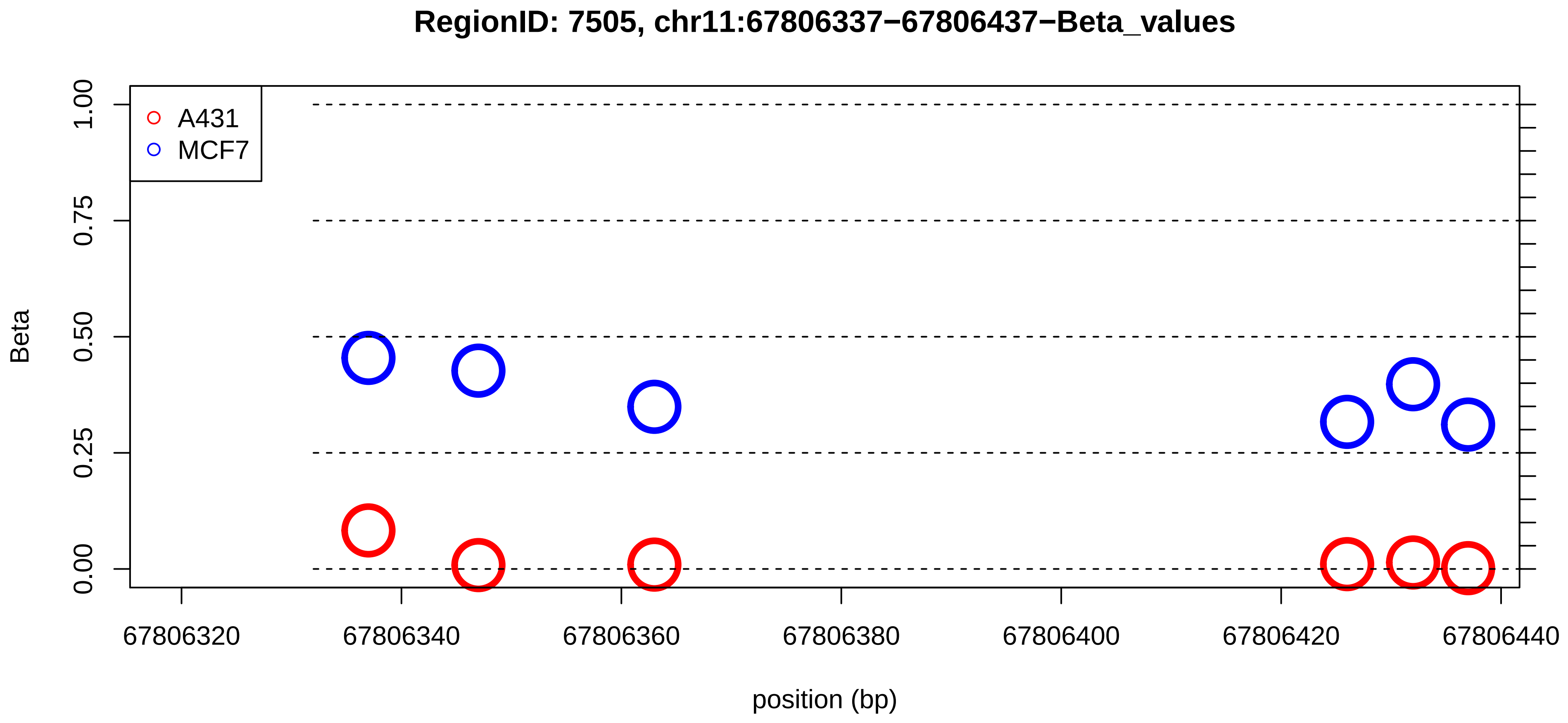
RegionID: 7505, chr11:67806337−67806437−Beta_values
A431
MCF7
Beta
1.00
0.75
0.50
0.25
0.00
67806320
67806340
67806360
67806380
67806400
67806420
67806440
position (bp)

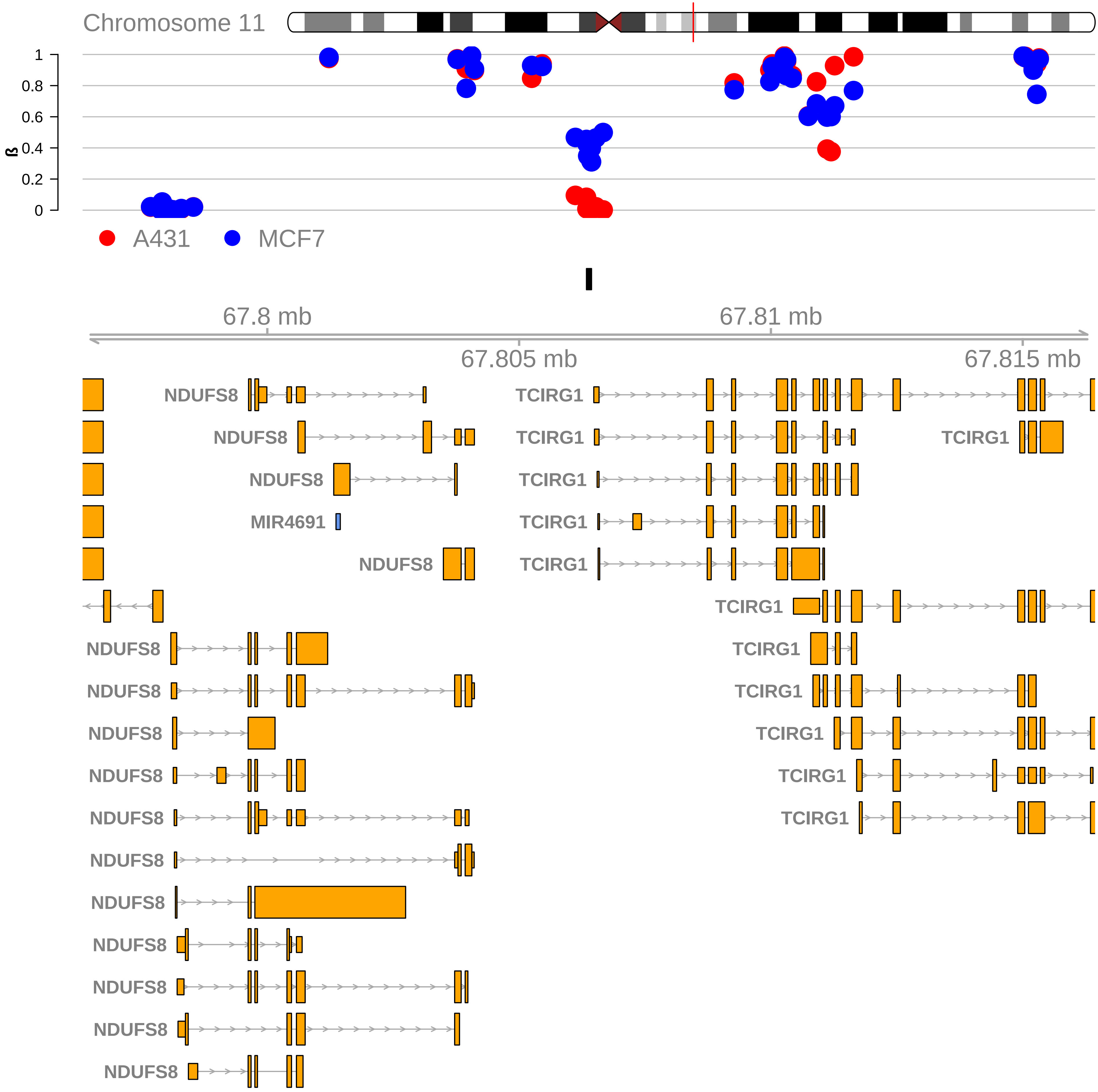

Chromosome 11
1
0.8
0.6
ß 0.4
0.2
0
A431
MCF7
67.8 mb
67.81 mb
67.805 mb
67.815 mb
NDUFS8
TCIRG1
NDUFS8
TCIRG1
TCIRG1
NDUFS8
TCIRG1
MIR4691
TCIRG1
NDUFS8
TCIRG1
TCIRG1
NDUFS8
TCIRG1
NDUFS8
TCIRG1
NDUFS8
TCIRG1
NDUFS8
TCIRG1
NDUFS8
TCIRG1
NDUFS8
NDUFS8
NDUFS8
NDUFS8
NDUFS8
NDUFS8
